# SFA-DETR: An Efficient UAV Detection Algorithm with Joint Spatial–Frequency-Domain Awareness

**DOI:** 10.3390/s25216719

**Published:** 2025-11-03

**Authors:** Peinan He, Xu Wang

**Affiliations:** College of Air Traffic Management, Civil Aviation Flight University of China, 46 Nanchang Road, Deyang 618307, China; wangxu@cafuc.edu.cn

**Keywords:** object detection, anti-UAV systems, RT-DETR

## Abstract

Unmanned Aerial Vehicle (UAV) detection often faces challenges such as small target size, loss of textural details, and interference from complex backgrounds. To address these issues, this paper proposes a novel object detection algorithm named Spatial-Frequency Aware DETR (SFA-DETR), which integrates both spatial- and frequency-domain perception. For spatial-domain modeling, a backbone network named IncepMix is designed to dynamically fuse multi-scale receptive field information, enhancing the model’s ability to capture contextual information while reducing computational cost. For frequency-domain modeling, a Frequency-Guided Attention Block (FGA Block) is introduced to improve perception of target boundaries through frequency-aware guidance, thereby increasing localization accuracy. Furthermore, an adaptive sparse attention mechanism is incorporated into AIFI to emphasize semantically critical information and suppress redundant features. Experiments conducted on the DUT Anti-UAV dataset demonstrate that SFA-DETR improves mAP50 and mAP50:95 by 1.2% and 1.7%, respectively, while reducing parameter count and computational cost by 14.44% and 3.34%. The results indicate that the proposed method achieves a balance between detection accuracy and computational efficiency, validating its effectiveness in UAV detection tasks.

## 1. Introduction

The rapid development of unmanned aerial vehicle technology has promoted its application in various industries [1,2,3], but it has also led to incidents such as illegal flight and malicious abuse [4,5]. This not only poses a potential threat to personal privacy, but also threatens public safety. Therefore, detection and monitoring technology for drones is becoming increasingly important. Visual inspection based on object detection technology can achieve more intuitive and accurate recognition of drones by capturing and analyzing their visual features.

Object detection technology based on deep learning has been extensively researched. Common object detection frameworks include Faster Regions with Convolutional Neural Networks (Faster R-CNN) [6] and You Only Look Once (YOLO) series [7]. These detection algorithms typically rely on Non-Maximum Suppression (NMS) operations to eliminate redundant prediction boxes for final detection results. However, with the application of Transformers in image processing, detection algorithms based on Transformer architectures have been proposed, such as Detection Transformer (DETR) [8]. By employing an object query mechanism, DETR eliminates the need for NMS, and its end-to-end detection paradigm has garnered increasing research attention. However, current object detection algorithms still have shortcomings in drone detection. For example, multiple downsampling is prone to the loss of shallow details in deep networks, leading to missed detections in small object detection at medium to long distances. Additionally, colors and textures in complex backgrounds are a form of interference that further exacerbates the risk of detection errors.

Torralba [9] proposed a method for improving object detection by analyzing scene context information to address the problem of small object detection. It demonstrated that object detection not only relies on the appearance features of the target itself, but also on the relationship between the target and the background as an important basis. Especially in small object detection, target information is easily lost in deep networks, resulting in a lack of sufficient semantic information. In this case, modeling the relationship between the target and context will provide richer discriminative criteria, guiding attention to make the right choices.

However, traditional detection algorithms mainly rely on stacking small convolution kernels. Although this can efficiently extract local information, its ability to perceive contextual information is relatively weak, which is not conducive to modeling the relationship between target and scene information in drone detection tasks. In the case where small-sized targets lose information due to downsampling, the relationship between the target and scene information will actually be more valuable for detection. Therefore, it is necessary to find new network mechanisms to obtain contextual information while avoiding additional computational costs.

In addition, mainstream detection algorithms often model features in the spatial domain, but under the interference of similar background information or a lack of significant texture features in the target, the processing methods in the spatial domain are prone to misjudgment. In this case, the contour and boundary of the target are more reliable features that can be utilized. Therefore, compared to spatial-domain methods such as Convolutional Neural Networks (CNNs) or Transformer networks for feature acquisition of the entire image, frequency-domain processing methods have more opportunities because they guide the model to perceive changes in the contours and boundaries of the target in the image to obtain target features. Specifically, high-frequency signals correspond to drastically changing boundaries, and low-frequency signals correspond to the subjects within the boundaries. This provides a new perspective for extracting image features for small-sized target detection.

Based on the above, this article proposes a new unmanned aerial vehicle detection algorithm, Spatial-Frequency Aware Detection Transformer (SFA-DETR), which perceives and obtains small-sized target features by using a joint processing mechanism of spatial and frequency domains designed to improve the performance of unmanned aerial vehicle detection in complex scenes. The main contributions of this study are as follows:

In terms of spatial-domain modeling, a new backbone network, IncepMix, is proposed by combining Inception parallel with multi-receptive field information. It improves the modeling ability of target and context by dynamically fusing information from different receptive fields, and reduces computational costs through channel compression strategy.

In terms of frequency-domain modeling, a Frequency-Guided Attention (FGA) Block was designed to guide the attention direction of the attention mechanism by utilizing the frequency information in the image, making it more inclined to focus on the boundary and contour information of the target, thereby improving the positioning accuracy.

We adopt an adaptive sparse attention mechanism [10] to replace the standard attention in the Attention-based Intra-scale Feature Interaction (AIFI), in order to highlight important semantics and suppress redundant interference.

## 2. Related Work

### 2.1. Object Detection Framework

The mainstream object detection algorithms are divided into two types, namely two-stage algorithms and one-stage algorithms. The two-stage algorithm first generates region proposals and then performs classification regression. Representative algorithms of this type include Regions with Convolutional Neural Networks (R-CNN) [11], Fast Regions with Convolutional Neural Networks (FastR-CNN) [12], and Faster R-CNN. The one-stage algorithm directly performs classification and regression on the feature map. Its representative algorithm is the YOLO series, which has the characteristics of simplicity and efficiency, and is therefore widely used in the research of unmanned aerial vehicle detection algorithms. Hao [13] introduced a P2 Detection Head based on YOLO v8 and designed a detail enhancement module to improve the detection accuracy of small targets, and introduced a multi-scale module in the neck to obtain multi-scale information. Cheng [14] promoted a dual receptive field module based on YOLO v8, which improved the robustness of the model in complex scenes. Gao [15] applied YOLO v11 as a benchmark and employed wavelet transform for downsampling in the backbone, reducing computational costs and improving the performance of the model in detecting small targets.

The development of Transformer [16] has brought about new detection architectures. Carion [8] proposed a Transformer-based detection framework DETR, which achieves end-to-end detection through a target query mechanism, avoiding post-processing designs such as non-maximum suppression. But it has not been widely used due to problems such as difficult convergence and poor performance in detecting small targets. Real-Time Detection Transformer (RT-DETR) [17] has been improved based on DETR. In the design of the encoder, it has been experimentally proven that simultaneous interaction at the same scale and across scales can result in a significant amount of computational redundancy and contribute less to the improvement of detection accuracy. Therefore, it claims that only exchanging information on the high-level semantics of the S5 layer is a more cost-effective solution. By using the Path Aggregation Network-like (PAN-like) scale fusion network CNN-based Cross-scale Feature Fusion (CCFF) to achieve cross-scale fusion, it can effectively improve the detection performance of multi-scale targets. In the target query mechanism, IOU (Intersection over Union)-aware query selection was created by introducing IOU constraints, which alleviated the problem of difficult convergence. In general, through the above solutions, RT-DETR has achieved fast and accurate detection while maintaining the advantages of DETR’s end-to-end architecture. In particular, it utilizes Transformer to achieve global information exchange, enhances global modeling capabilities, and fills a new architectural solution for unmanned aerial vehicle detection in complex environments.

### 2.2. Development of Spatial-Domain Feature Extraction Methods

The spatial-domain method models the spatial structure of an image at the pixel level or feature layer, and is the main way to extract features from images. Convolutional Neural Networks (CNNs) and Transformer networks have been widely used. Since the introduction of AlexNet [18], CNNs have become the main tool for image processing in deep learning, effectively applied to computer vision related tasks by gradually downsampling and extracting high-dimensional features from input images. Afterwards, two main architectures were developed, namely Inception [19] and Visual Geometry Group Network (VGG Net) [20]. Inception employs a method of parallel feature extraction using multiple large and small convolution kernels, and then fusing these features of different scales. This method can obtain larger receptive field information, but the use of large convolution kernels also brings significant computational complexity. VGG Net uses multiple small convolution kernels to continuously deepen the network, which requires less computation compared to large-convolution kernels in terms of performance. It also demonstrates that replacing large convolution kernels with multiple small convolution kernels is more efficient, which has become the mainstream design concept for future architectures.

However, the stacking of small convolutional kernels does not effectively expand the receptive field in practice, resulting in limitations for image processing models in capturing long-range dependencies. To enhance global modeling capabilities, researchers integrated Transformers with CNNs, leading to hybrid CNN + Transformer approaches. Later, pure Transformer-based backbone networks such as Vision Transformer (ViT) [21] and Swin Transformer [22] were proposed.

However, the use of Transformers often comes with substantial computational costs. To address this efficiency challenge, researchers have proposed various optimization strategies. Wei [23] introduced a pruning approach for ViT layers, noting that shallow features undergo significant transformations while deeper features tend to saturate. Their method progressively prunes redundant layers from deep to shallow, ultimately achieving an optimal balance between performance and speed. Similarly focusing on deep-layer optimization, Xue [24] proposed disabling highly similar layers in deeper networks while retaining only the optimal ones, thereby maintaining accuracy while improving efficiency. Wu [25] designed an adaptive activation module that selectively activates Transformer blocks based on the complexity of the feature information being processed, thereby enhancing the computational efficiency of ViT architectures.

Following the introduction of ViT, researchers revisited the potential of CNNs in global information processing and turned their attention back to large-kernel convolution. The proposal of dilated convolutions [26] alleviated computational concerns by expanding the receptive field without increasing computational costs. RepLKNet [27] was the first to employ a design based on large-kernel convolutional neural networks (ConvNets), which adopted the architecture of Swin Transformer and successfully improved the receptive field of the model. Building on RepLKNet, UniRepLKNet [28] reconstructed the architecture of a super large-kernel convolutional neural network, making it superior to Swin Transformer in terms of performance and inference speed, demonstrating that well-designed large-kernel convolutions can match the effectiveness of Transformers, although still at the cost of a large computational load.

To avoid the limitation of a single receptive field in drone detection, it is necessary to introduce both local information from small convolution kernels and contextual information from large convolution kernels. With the development of large convolution kernels, the parallel convolution architecture embodied in Inception provides another area of optimization research. This article introduces a method that combines UniRepLKNet and Inception-v4 [29] to construct a new backbone network. By dynamically fusing multi-scale receptive field information, richer contextual information is obtained to guide attention selection. Progressive channel expansion is used to significantly reduce the computational load of large convolution kernels.

### 2.3. Research Based on Frequency-Domain Methods

YU [30] disassembled Transformer into two parts: MetaFormer and Token Mixer, and demonstrated through experiments that the MetaFormer structure is the key to the success of Transformer. From then on, researchers began to consider how to find more efficient Token Mixers within the MetaFormer structure, rather than relying solely on attention mechanisms to achieve global information exchange. A prominent development in this process is the use of frequency-domain processing methods.

Tatsunami [31] transformed spatial information into the frequency domain using Fast Fourier Transform (FFT) and designed a dynamic filter that can dynamically adapt to high-frequency and low-frequency information, reducing information loss. GAO [32] divides information into two modules, spatial domain and frequency domain, through FFT for simultaneous computation. The spatial details and global information in the time domain are used to complement each other, and this design is applied to image restoration. On this same application, referring to the structural design of Transformer, Jiang [33] proposed SFHformer, which uses FFT to obtain global information and designs local information extraction, and further processes the features of both information through CNN network.

The above methods usually adopt frequency-domain methods to obtain global feature relationships in image processing, and design local feature extraction modules to supplement spatial information. In object detection, a frequency-domain guidance method can guide the model to perceive boundary features in the image. Based on this, this paper applies a frequency-domain processing method to unmanned aerial vehicle detection and proposes a more concise frequency-domain guidance module, which is more in line with the general architecture of object detection algorithms, strengthens the modeling ability of the boundary between the target and the background, and improves the positioning accuracy and interference suppression ability.

## 3. Proposed Method

### 3.1. Overall Architecture

RT-DETR employs a CNN backbone for feature extraction, followed by a Transformer module at the network’s terminus to enable global information interaction. The Cross-scale Feature Fusion (CCFF) module integrates multi-level features from the backbone, with its output subsequently fed into the decoder. The model achieves end-to-end detection through an object query mechanism. To accelerate training, an IOU-aware query selection strategy is incorporated, introducing IOU constraints during training to guide the queries toward higher-quality candidate regions.

**Figure 1:**
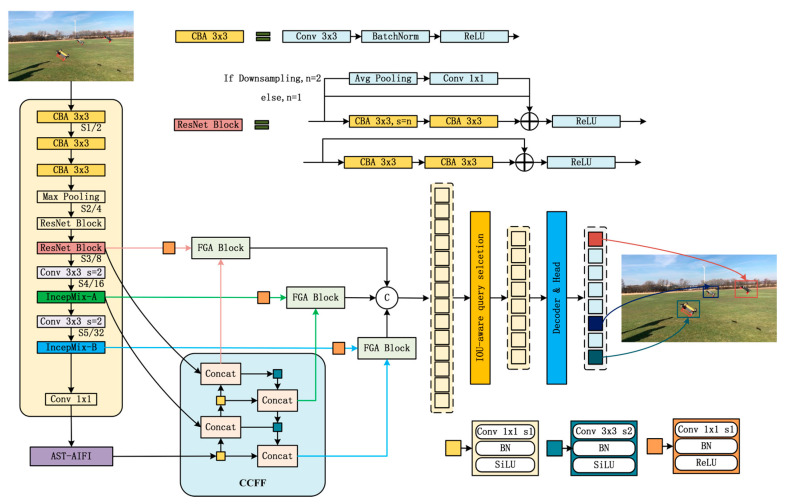
Architecture of Spatial-Frequency Aware DETR (SFA-DETR).

To further enhance drone detection performance, on the basis of RT-DETR, this paper proposes a detection algorithm based on spatial–frequency joint sensing: Spatial-Frequency Aware DETR (SFA-DETR). Its overall architecture is shown in Figure 1. This method combines the advantages of the spatial-domain method and frequency-domain processing method to obtain richer perceptual information to guide the model to focus on key areas. In the aspect of spatial-domain modeling, this paper proposes the backbone network IncepMix, which helps the model make more effective use of context clues by dynamically fusing multi-scale receptive field information, so as to enhance the detection ability; In the aspect of frequency-domain modeling, a frequency-guided attention block (FGA block) is proposed, which integrates the shallow features of the trunk and the semantic features of CCFF. By extracting multi-level frequency information, the perception of edge changes of the model is enhanced, so as to improve the discrimination ability of the model under the interference of background information. Finally, the adaptive sparse attention mechanism is introduced to replace the standard attention mechanism of AIFI, highlighting the key semantic information and suppressing the interference of redundant information.

### 3.2. IncepMix Network Design

To acquire richer receptive field information and enhance the model’s detection capability, this paper proposes the IncepMix network, incorporating Inception’s design philosophy of parallel multi-branch fusion. Specifically, for the S3 layer, which serves as a shallow feature layer inclined to capture local information such as detailed textures, ResNet [34] is adopted to fully preserve critical detailed information. For the S4 and S5 layers, two substructures—IncepMix-A and IncepMix-B—are designed, respectively. By integrating information from different receptive fields, these substructures enhance the model’s ability for multi-scale perception and contextual understanding.

1. CSP-UniRepLKNet Block

**Figure 2:**
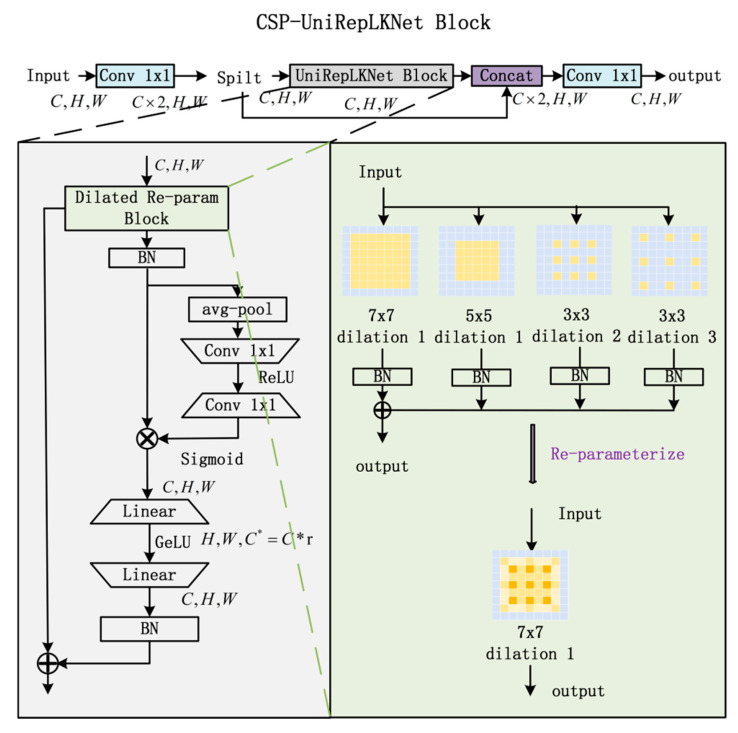
Architecture of CSP-UniRepLKNet Block.

UniRepLKNet module is introduced to capture broader receptive field information. By integrating the design concept of Cross-Stage Partial Network (CSP), the CSP-UniRepLKNet block is proposed to enhance stability and efficiency, as illustrated in Figure 2. UniRepLKNet employs a dilated reparameterization architecture, which improves representational capacity through parallel integration of large-kernel convolutions and small-scale dilated convolutions. Channel-wise attention and spatial feature aggregation are subsequently achieved using a Squeeze-and-Excitation (SE) mechanism followed by nonlinear transformation.

2. IncepMix

**Figure 3:**
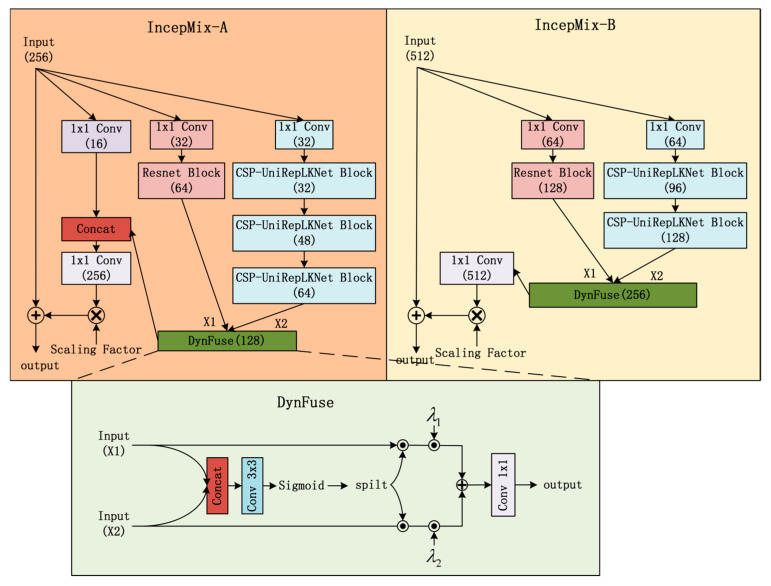
Architectures of IncepMix-A and IncepMix-B.

As shown in Figure 3, IncepMix incorporates CSP-UniRepLKNet to capture contextual information and uses ResNet for extracting local features. The channel count of each branch is reduced to decrease computational cost. A progressive channel expansion strategy is applied in the large receptive field branch to enhance feature modeling layer by layer. Feature fusion from the two branches is dynamically performed via a DynFuse module. DynFuse conducts weighted fusion of the two branches’ outputs using attention-generated weights and trainable parameters, as expressed in Equation (1):

(1) $$\left\{\begin{array}{c} \left[\alpha_{1},\alpha_{2}]=spilt(sigmoid(Con\nu_{3\times 3}(Concat(X_{1},X_{2})))\right)\\ output=Con\nu_{1\times 1}(\lambda_{1}⊙(\alpha_{1}⊙X_{1})+\lambda_{2}⊙(\alpha_{2}⊙X_{2})) \end{array}\right.$$

Here, $\lambda_{1},\lambda_{2}$ are trainable weights, and ⊙ denotes element-wise multiplication. The fused features are scaled by a factor and then integrated with the input via a residual connection.

Based on the functional characteristics of different stages in the backbone network, IncepMix-A and IncepMix-B are specifically designed for the S4 and S5 layers, respectively. Considering that the S4 layer retains both spatial and semantic information, IncepMix-A incorporates a lightweight 1 × 1 convolutional branch to preserve the original feature structure and facilitate the transmission of fine-grained details. In contrast, as the S5 layer is primarily responsible for high-level semantic modeling, IncepMix-B employs a more compact channel expansion strategy to reduce computational redundancy and omits the 1 × 1 convolutional branch to focus exclusively on processing advanced semantics.

### 3.3. Design of Frequency-Guided Mechanism

**Figure 4:**
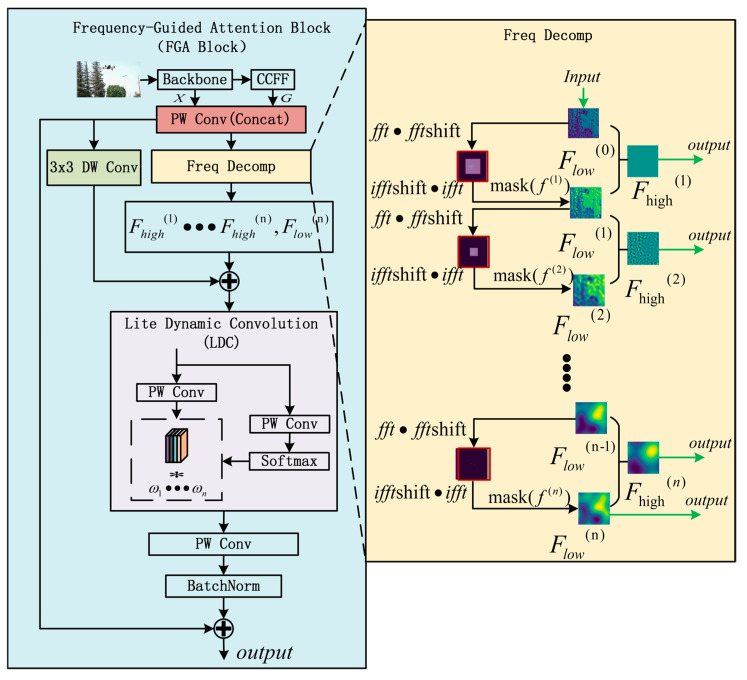
Architecture of the Frequency-Guided Attention Block (FGA Block).

To address the challenges of distinguishing object–background boundaries and mitigating interference from similar background patterns, this paper proposes the Frequency-Guided Attention Block (FGA Block), a novel module that enhances localization precision through frequency-domain processing. As illustrated in Figure 4, the approach integrates shallow features from the backbone network with Cross-level Context Fusion Features (CCFF) to preserve fine-grained details of small objects in deep layers while maintaining semantic richness from deep features.

The core of the method involves multi-level frequency information extraction via Fast Fourier Transform (FFT). Leveraging the non-trainable nature of FFT, the model maintains computational efficiency by avoiding additional gating mechanisms on frequency components. Since frequency representations lack spatial information, Depthwise Separable Convolution (DW Conv) is employed outside the frequency domain to capture spatial relationships. A lightweight channel attention-based dynamic convolution is subsequently applied to emphasize informative channels.

Extensive ablation studies in Section 4.4.2 validate the effectiveness of the architectural design. The detailed algorithm proceeds as follows:

The FGA Block takes two feature maps as inputs: $X\in R^{B\times C_{1}\times H\times W}$ from the backbone network and $G\in R^{B\times C_{2}\times H\times W}$ from the CCFF module, where *B* denotes batch size, H×W represents spatial dimensions, and $C_{1},C_{2}$ indicates channel numbers. These features are concatenated and integrated through a fusion operation to produce feature map $F_{in}$.

1. Frequency Mask Construction and Multi-level Frequency Extraction

A frequency list $F=\{f_{1},f_{2},...,f_{n}\}$ is defined where parameter $f_{i}\in R^{+},\;f_{i}>1$ controls the mask size at different frequency levels. Notably, larger values of $f_{i}$ produce smaller masks. Each mask $M_{h,w}^{\left(i\right)}$ operates by preserving information from the central image region while suppressing peripheral areas, enabling focused frequency-domain processing, as expressed in Equation (2):

(2) $$M_{h,w}^{\left(i\right)}=\left\{\begin{array}{c} 1,\left|h-\frac{H}{2}\right|\leq \frac{H}{2f_{i}},\left|w-\frac{w}{2}\right|\leq \frac{w}{2f_{i}}\\ 0,otherwise \end{array}\right.$$

The input feature map $F_{in}$ is first transformed into the frequency domain via Fast Fourier Transform (FFT), with low-frequency components shifted to the centre. Mask $M_{h,w}^{\left(i\right)}$ is applied to extract the low-frequency information at the (i−1) level. The high-frequency information at this level is obtained by subtracting the i level low-frequency components from the (i−1) level features. Finally, multi-level high-frequency information along with the low-frequency $F_{low}$ information from the final level are aggregated to produce the output representation $F_{freq}$, as expressed in Equation (3):

(3) $$\left\{\begin{array}{c} F_{low}^{\left(0\right)}=F_{in}\\ F_{masked}^{\left(i\right)}=FFTShift\left(FFT\left(F_{low}^{\left(i-1\right)}\right)\right)\cdot M^{\left(i\right)}\\ F_{low}^{\left(i\right)}=iFFT\left(iFFTShift\left(F_{masked}^{\left(i\right)}\right)\right)\\ F_{high}^{\left(i\right)}=F_{low}^{\left(i-1\right)}-F_{low}^{\left(i\right)}\\ F_{low}=F_{low}^{\left(n\right)}\\ F_{freq}=\sum_{i=1}^{n}\;F_{high}^{\left(i\right)}+F_{low}^{\left(n\right)} \end{array}\right.$$

2. Spatial information enhancement and feature aggregation

Perform Depthwise Separable Convolution on $F_{in}$ to introduce spatial information, as shown in Formula (4):

(4) $$F_{spatial}=DWCon\nu_{3\times 3}\left(F_{in}\right)$$

Fuse frequency-domain features with spatial information, as shown in Formula (5):

(5) $$F_{multi}=F_{spatial}+F_{freq}$$

3. Lightweight Dynamic Convolution (LDC)

Lightweight dynamic convolution is applied to $F_{multi}$ with channel-wise weighting mechanism. Firstly, the number of channels is compressed to n using Point-wise Convolution (PW Conv), with one branch generating feature responses and the other branch generating channel-wise weighting using softmax. Thus, more important channel information can be selected at each spatial position to suppress redundant features and achieve dynamic channel weighting, as shown in Formula (6). Finally, connect the output ${\tilde{f}}_{dc}^{\left(u,v\right)}$ with $F_{in}$ to maintain the flow of the original information.

(6) $${\tilde{f}}_{dc}^{\left(u,v\right)}=\sum_{i=1}^{n}\;w_{i}^{\left(u,v\right)}\cdot conv_{i}^{\left(u,v\right)}$$

Here, $\left(w_{1}^{\left(u,v\right)},...,w_{n}^{\left(u,v\right)}\right)$ represents the attention weight of the *i*-th channel at position (u,v), and $conv_{i}^{\left(u,v\right)}$ represents the feature map value of the *i*-th channel at that position.

### 3.4. Adaptive Sparse Attention Mechanism

**Figure 5:**
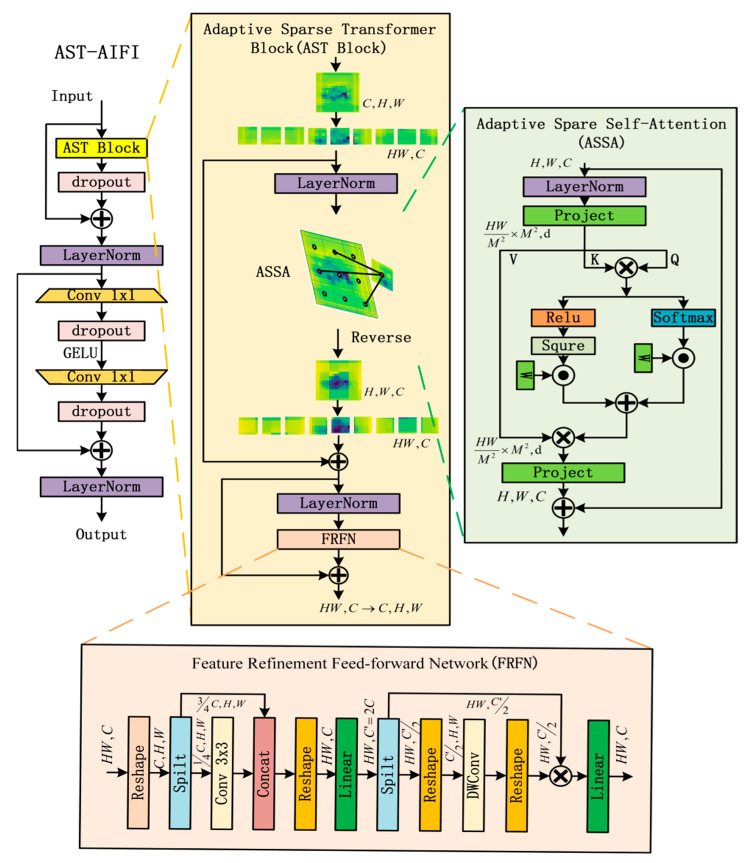
Architecture of the AST-AIFI Module.

AIFI employs the standard attention mechanism to enable information interaction between features. However, in UAV detection tasks, interactions among all tokens may introduce redundant information from non-critical regions, diluting the response of key areas and thus degrading detection accuracy. To mitigate this issue, this paper replaces the standard attention mechanism in AIFI with an adaptive sparse attention mechanism and designs AST-AIFI, whose structure is illustrated in Figure 5. The input feature map is processed through Adaptive Sparse Self-Attention (ASSA) to capture global contextual relationships and highlight critical spatial information. The Feature Refinement Feed-forward Network (FRFN) then transforms the features into the $X\in R^{C,H,W}$ for convolutional operations, extracting channel-wise information features. Finally, a feed-forward network is applied to further enhance the feature representation capability. The computational process of ASSA is as follows:

1. The input feature map is reshaped into $X\in R^{C,H,W}$, which is then divided into non-overlapping windows of size M×M. For each window, the feature representation $X_{i}\in R^{M^{2}\times C}$ is obtained. From this, the query matrix Q, key matrix K, and value matrix V are generated. The attention is computed as:
  (7) $$A=f(QK^{T}/\sqrt{d}+B)V$$
  where A denotes the final attention output, B represents a learnable relative position bias, and f(·) refers to the scoring function.
2. Feature interaction is performed via Sparse Attention (SSA) and Dense Attention (DSA), as shown in Equations (8) and (9), respectively. SSA suppresses negative values through squared ReLU-based sparsification, filtering out query-key pairs with low matching scores while highlighting salient correlations. To prevent potential information loss, DSA facilitates global interaction among all elements via softmax, emphasizing critical information while suppressing less important components.

(8) $$SSA={ReLU}^{2}\left(QK^{T}/\sqrt{d}+B\right)$$

(9) $$DSA=SoftMax(QK^{T}/\sqrt{d}+B)$$

The outputs of SSA and DSA are fused using learnable weights, as defined in Equation (10):

(10) $$\left\{\begin{array}{c} A=\left(w_{1}\cdot SSA+w_{2}\cdot DSA\right)V\\ w_{n}=e^{a_{n}}/\sum_{i=1}^{N}\;e^{a_{i}},n=1,2 \end{array}\right.$$

Here, $w_{1},\;w_{2}$ are normalized weights, and $a_{1},a_{2}$ are learnable parameters.

## 4. Experimental Analysis

### 4.1. Dataset

This study primarily employs the DUT Anti-UAV [35] dataset for experimental evaluation. Proposed by a research team from Dalian University of Technology, this dataset is a visible-light anti-UAV detection dataset comprising 10,000 images. It is characterized by a high proportion of small objects, diverse types of drones, and complex detection environments, making it suitable for evaluating algorithm performance in challenging scenarios for UAV detection.

### 4.2. Experimental Environment and Parameter Settings

All experiments are conducted on a workstation equipped with an RTX 3090 GPU (24 GB VRAM) and an Intel^®^ Xeon^®^ E5-2698 v4 CPU. Other relevant parameter configurations are provided in Table 1.

### 4.3. Evaluation Metrics

Multiple metrics are adopted to evaluate model performance, including Precision, Recall, mean Average Precision (mAP), computational complexity (GFLOPs), number of parameters (Parameters), and inference speed (FPS). Among these, mAP50:95 provides a more comprehensive reflection of localization accuracy compared to mAP50. Based on mAP50:95, the results are further categorized into APS, APM, and APL, representing the accuracy for small-, medium-, and large-scale targets, respectively.

### 4.4. Ablation Studies

#### 4.4.1. Ablation Study on IncepMix

1. Ablation on the IncepMix Architecture

**Figure 6:**
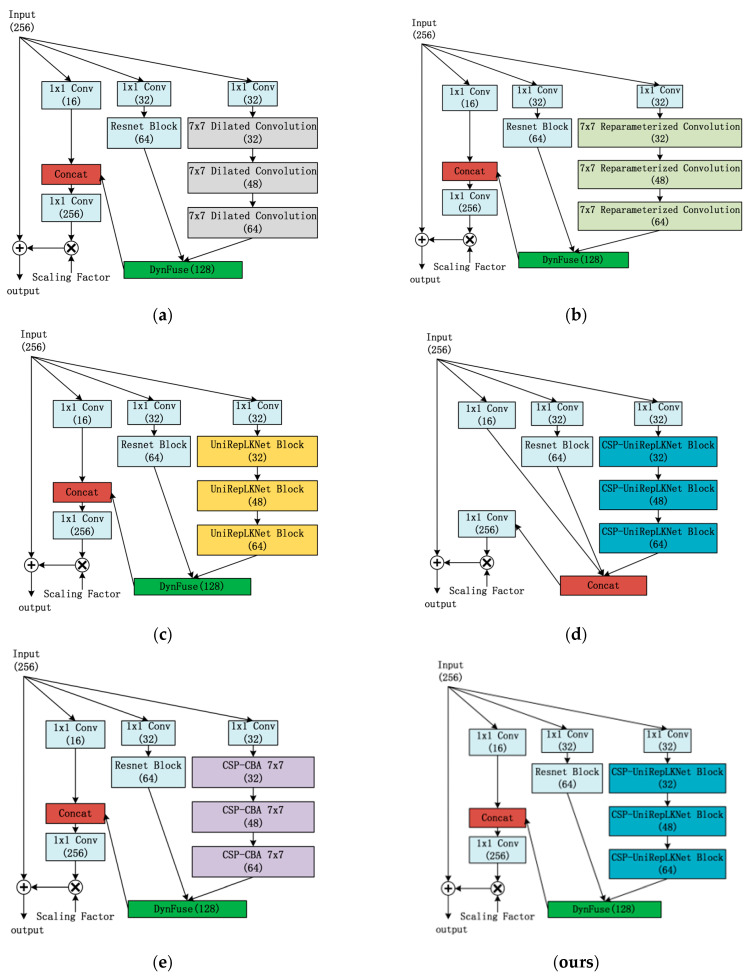
Ablation experiment of IncepMix structure; (**a**–**c**) Large convolution kernels using dilated convolution, reparameterized convolution, and UniRepLKBlock, respectively; (**d**) Removing DynFuse and using Concat for fusion; (**e**) Replacing CSP-UniRepLKBlock with CSP-CBA 7 × 7; (**ours**) The complete IncepMix structure proposed in this paper.

To validate the effectiveness of the IncepMix structural design, multiple architectural variants were developed for ablation studies, as illustrated in Figure 6. Using IncepMix-A as an example in the figure, IncepMix-B follows a corresponding design. The variants include (a)–(c) employing dilated convolution, reparameterized convolution, and UniRepLKNet, respectively, in the large-kernel branch; (d) removing the DynFuse module while concatenating all branches; (e) replacing UniRepLKNet with a standard 7 × 7 convolution enhanced by BatchNorm and activation functions to improve nonlinear representation capability. Following common practices in Inception architectures, the scaling factor in our experiments was set to 0.1. Experimental results are presented in Table 2.

Results from variants (a)–(c) demonstrate that UniRepLKNet contributes more significantly to accuracy improvement within our proposed framework, while the CSP structure shows greater benefits for mAP50:95 enhancement. After removing the DynFuse module (variant d), mAP50 decreases by 0.8% and mAP50:95 drops by 1.0%, yet the parameter count is reduced by 20.7% and computational load decreases by 8.92%, representing a cost-effective solution for resource-constrained scenarios. Experiment (e) reveals that the feature representation capability of standard 7 × 7 convolution is inferior to UniRepLKNet, resulting in significant performance degradation. Comprehensive experimental evaluation confirms that the complete IncepMix architecture achieves optimal detection performance.

Referring to common practices in Inception architectures, a scaling factor of 0.1 is used in the experiments. Results are shown in Table 2.

From experiment (a), it is observed that the CSP structure contributes to improved mAP50:95. In experiment (b), removing DynFuse leads to a decrease of 0.8% in mAP50 and 1% in mAP50:95, while reducing parameters by 38.34% and computational cost by 8.24%. This configuration offers a cost-effective trade-off under limited computational resources. Experiment (c) indicates that replacing UniReplKNet with a standard 7 × 7 convolution results in significant performance degradation, confirming the superiority of UniReplKNet in feature representation. Overall, the complete IncepMix structure achieves the best detection accuracy.

2. Impact of Kernel Size in UniRepLKNet

To further investigate the influence of the kernel size KK in UniRepLKNet within the IncepMix module, experiments are conducted with kernel sizes K∈{7, 9, 13, 17}. The results are presented in Table 3. The highest accuracy is achieved when K=7. Further increasing the kernel size does not yield additional performance improvements.

#### 4.4.2. Ablation Study on FGA Block

1. Impact of Different Frequency Masks

To investigate the detection performance of the FGA Block under different frequency masks, experiments are conducted with various sets of frequency values FF, as shown in Table 4. Results indicate that when F={2, 4}, the highest mAP50 is achieved. With F={4, 8}, better localization accuracy (mAP50:95) is obtained. However, when F={2, 4, 8}, mAP50:95 decreases significantly, suggesting that extracting an excessive number of frequency bands may introduce redundant high-frequency noise, which interferes with the model’s ability to discern critical edge information. Based on these findings, F={4, 8} is adopted in subsequent experiments.

2. Ablation Study on FGA Block Architecture

To validate the effectiveness of the FGA Block design, a series of experiments are conducted by incrementally adding its constituent modules, as summarized in Table 5. Here, S1 refers to the baseline model without introduced backbone features or improvements; “Concat” denotes the channel-wise concatenation of backbone and CCFF features. Models S2 to S9 are constructed by progressively integrating additional modules on top of the backbone and CCFF features.

Comparisons between S1 and S2 show that directly concatenating backbone and CCFF features without guided integration may introduce redundant information, thereby harming object localization. S4 and S6, which incorporate spatial information modeling, exhibit noticeable accuracy improvements over S2. By comparing S2, S3, S6, S8, and S9, it is observed that incorporating frequency-domain guidance enhances localization accuracy. The highest performance is achieved when spatial information is supplemented and non-critical channel information is suppressed via a channel attention weighting mechanism. These results demonstrate that the FGA Block, through the synergistic effect of its submodules, brings significant performance gains, confirming the efficiency and effectiveness of the proposed design.

#### 4.4.3. Ablation Study on Overall Modules

To evaluate the impact of each proposed improvement on the model, ablation studies were conducted by incrementally incorporating the modifications using the DUT Anti-UAV dataset. The RT-DETR model with a ResNet-18 backbone was selected as the baseline. In the experiments, Component A denotes replacing the backbone network with IncepMix, Component B represents incorporating feature information from both the backbone network and CCFF along with utilizing the FGA Block, and Component C indicates replacing the original AIFI module in the baseline model with AST-AIFI. The experimental results are summarized in Table 6. Furthermore, to comprehensively assess the effect of each improvement on targets of different scales, Table 7 presents the detection accuracy for small-, medium-, and large-sized targets achieved by the respective modifications.

Based on the experimental results, the following observations can be drawn:

1. Modules A, B, and C each enhance model performance from different perspectives. Module A improves contextual modeling through its multi-branch receptive field design, increasing mAP50 by 0.6% and mAP50:95 by 0.5%, with performance gains observed across all target scales. Owing to its channel compression mechanism, the number of parameters is reduced by 22.24%, computational cost (GFLOPs) decreases by 10.37%, and FPS improves by 12%. Module B incorporates frequency-domain processing, strengthening the model’s perception of edge structures. This leads to improvements in both precision and recall, albeit at the cost of increased computational overhead. Module C enhances semantic representation to improve detection of small targets, achieving the highest APs of 63.2%. However, its effect on medium-scale targets remains limited.
2. The A + B combination leverages synergistic modeling of contextual spatial information and frequency-domain structural features, raising mAP50 to 96.8% and recall to 95.2%, approaching the final performance level. This indicates that the model detects more objects through joint spatial–frequency modeling, though boundary perception is not substantially enhanced, and mAP50:95 sees no further improvement. The B + C combination emphasizes the collaboration between frequency-domain methods and global semantic interaction. Module C suppresses interference from redundant information at the semantic level, mitigating noise introduced by frequency processing and thereby improving localization accuracy and precision. This configuration achieves the highest mAP50:95 of 71.1% and the best performance on large targets with APL reaching 79.5%.
3. It is noteworthy that although the A + C combination improves precision, it leads to a noticeable decline in both mAP50:95 and recall. This suggests that while multi-scale fusion in A enhances perception of salient regions and Module C further suppresses redundant information—effectively reducing false detections—it also weakens boundary and detail representation, resulting in reduced localization accuracy. After incorporating Module B, shallow backbone features are introduced to compensate for the loss of local information, and frequency-guided enhancement helps restore boundary detection capability, addressing the limitation in localization precision.

With all three modules integrated, mAP50 improves by 1.2%, mAP50:95 increases by 1.7%, the number of parameters is reduced by 14.44%, and computational cost decreases by 3.34%. The results demonstrate that the proposed improvements complement each other effectively, collectively achieving the best overall performance.

**Figure 7:**
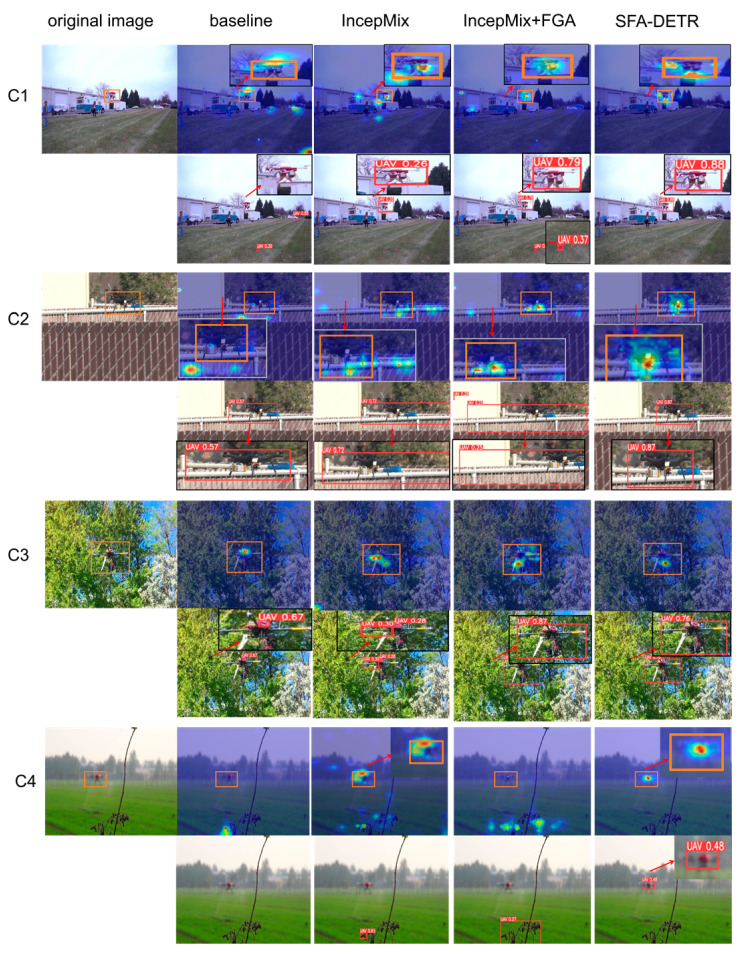
The detection performance on three representative UAV scenarios (**C1**–**C4**) is illustrated. Each group presents the original image, along with the heatmaps and detection results under the improvements of IncepMix, IncepMix + FGA, and the final SFA-DETR. The heatmaps are used to visualize the regions attended by the model during the detection process, where red indicates high-response areas and blue indicates low-response areas.

To further validate the performance of the proposed method in UAV detection, visual analysis was conducted using HiResCAM and detection results, as shown in Figure 7:

1. In scenario C1, the baseline model suffers from interference caused by similar background textures, leading to dispersed attention and resulting in missed detections and false alarms. After incorporating IncepMix to capture broader contextual information, the model begins to focus more on the target, but the attention remains imprecise, resulting in low detection confidence. With the further addition of the FGA Block, the model shows enhanced attention to target contours, albeit with slight dispersion and occasional false detections. Finally, after integrating AST-AIFI, the adaptive sparse attention mechanism effectively suppresses redundant information, enabling the model to concentrate more accurately on the target and achieve high-confidence detection results.
2. In scenario C2, due to the similar textures between the target and the background, as well as high-frequency boundary interference, the first three detection configurations exhibit noticeable localization shifts and low confidence. Leveraging frequency-domain guidance and semantic enhancement mechanisms, SFA-DETR effectively captures the structural information of the target, achieving high-confidence detection with precise boundary localization.
3. In scenario C3, the presence of dense and similar high-frequency textures in the background makes it difficult for the baseline model to perceive boundary information, leading to misidentification of partial regions as the entire target. After adding IncepMix, contextual information is incorporated, partially reducing false detections, but the model still fails to detect the complete target. The further integration of the FGA Block significantly improves the model’s attention to boundaries, enabling accurate localization. However, with the subsequent addition of AST-AIFI, although certain redundant information is successfully suppressed, the sparse attention mechanism incorrectly dilutes some critical boundary information due to the similar high-frequency textures between the target and the background, resulting in a slight decrease in detection confidence. This indicates that the model’s ability to distinguish high-frequency details in such cluttered environments requires further optimization.
4. In Scenario C4, where the image focuses on branches, the drone target appears blurred with distorted boundary and texture information. The baseline model fails to recognize the drone, leading to a misdetection. After incorporating IncepMix, the model begins to notice the target but remains susceptible to branch interference, resulting in continued misjudgment. With the further addition of the FGA module, the more prominent high-frequency signals from branches divert the model’s attention toward them. Finally, after integrating the AST-AIFI module, the interference from branches is suppressed, allowing the model to refocus on the drone target. However, due to the excessively blurred boundary information of the drone, the localization accuracy remains poor, and the confidence score is low. These observations indicate that when target boundaries and texture features are severely degraded, the detection performance of the model still requires further optimization.

In summary, the experiments demonstrate that IncepMix, FGA Block, and AST-AIFI work in a complementary and synergistic manner, enhancing contextual modeling, boundary perception, and redundancy suppression. The proposed method effectively improves detection performance in scenarios involving small targets and complex background interference, validating its overall effectiveness.

### 4.5. Comparative Experiments

1. Comparison with Real-time Detection Algorithms

To evaluate the overall performance of the proposed SFA-DETR model against mainstream detection methods, comparative experiments were conducted on the DUT Anti-UAV dataset using widely adopted real-time detection algorithms. The results are summarized in Table 8. Experimental results demonstrate that SFA-DETR achieves superior accuracy with fewer parameters and lower computational cost.

2. Generalization Capability Comparison Experiments

To validate the generalization capability of the proposed SFA-DETR algorithm, comparative experiments were conducted using the drone datasets Det-Fly [36] and Anti2 [37]. Det-Fly, captured by another drone during flight, contains over 13,000 images divided into “simple” and “complex” subsets based on detection difficulty. The complex subset features smaller targets and more background interference. The Anti2 dataset comprises 5062 images covering multiple scenarios and introduces three different types of flying objects—airplanes, helicopters, and birds—to test the impact of interference from other flying objects on drone detection accuracy in real-world detection environments.

The experimental results are presented in Table 9. The results demonstrate that on the complex subset of Det-Fly, the improved SFA-DETR model achieves a 1.3% increase in mAP50 and a 3.2% increase in mAP50:95. On the Anti2 dataset, the improved model shows a 1.2% gain in mAP50 and a 3.1% improvement in mAP50:95. These experimental findings indicate that the proposed SFA-DETR algorithm possesses strong generalization capability, with particularly notable accuracy improvements in more challenging detection scenarios.

## 5. Conclusions

To address the challenges of detecting small UAV targets susceptible to background interference in complex environments, this paper proposes SFA-DETR, a detection algorithm based on joint spatial–frequency-domain perception. Built upon the RT-DETR architecture, the method enhances model perception from both spatial and frequency domains.

For spatial modeling, the IncepMix backbone network is designed to dynamically integrate multi-scale receptive field information, effectively improving contextual understanding while reducing computational overhead through a channel compression strategy. For frequency-domain modeling, the Frequency-Guided Attention (FGA) Block is introduced, which incorporates both backbone and CCFF features to capture local and global semantics. By extracting multi-level high- and low-frequency information and applying lightweight dynamic convolution based on channel attention, the model more accurately focuses on target boundaries and contours, enhancing detection performance in cluttered backgrounds. Additionally, an adaptive sparse attention mechanism is integrated into AIFI to emphasize critical semantics and suppress redundant information, further improving detection accuracy.

Experimental results validate that the proposed improvements work synergistically, boosting the model’s detection capability in challenging environments. However, the proposed method still has certain limitations. When the background contains dense high-frequency patterns similar to the target, distinguishing object boundaries becomes difficult, and the adaptive sparse attention may incorrectly dilute crucial edge information, reducing detection performance. When the texture or boundary information of the target is severely degraded, the localization accuracy may also be compromised. Future work will focus on enhancing the model’s boundary perception ability and strengthening the collaboration between spatial and frequency modeling to improve both detection accuracy and overall robustness.

## Figures and Tables

**Table 1:** Experimental Settings.

Parameters	Setup
Datasets	DUT Anti-UAV, Det-Fly
Epochs	150
Train Batch Size	8
Test Batch Size	16
Image Size	640
Workers	4
Optimizer	AdamW
lr0	0.0001
Momentum	0.9
Weight decay	0.0001

**Table 2:** Ablation Results of the IncepMix Structure.

Variant	mAP50/%	mAP50:95/%	Params/M	GFLOPs	FPS
a	96.7	68.9	16.48	52.4	149
b	96	69.7	15.4	50.9	160
c	96.3	70.1	15.64	51.3	132
d	95.5	69.5	12.25	46.8	143
e	95.3	69.7	15.78	51.5	116
ours	96.3	70.5	15.45	51	133

**Table 3:** Ablation Study on the Kernel Size of UniRepLKNet in IncepMix.

K	mAP50/%	mAP50:95/%	Params/M	GFLOPs
K=7	96.3	70.5	15.45	51
K=9	95.5	70	15.46	51.1
K=13	96	70.2	15.5	51.1
K=17	95.4	69.9	15.5	51.1

**Table 4:** Impact of Different Frequency Mask Configurations on FGA Block Performance.

F	mAP50/%	mAP50:95/%	FPS
2	96.5	70.6	99
4	96.5	70.6	116
2, 4	96.8	69.6	108
4, 8	96.4	70.8	110
2, 4, 8	96.5	69.2	102

**Table 5:** Experimental Results of FGA Block Structure Effectiveness.

Structure	Concat	Freq Decomp	DW Conv	LDC	mAP50/%	mAP50:95/%	GFLOPs
S1					95.7	70	56.9
S2	**√**				95.9	68.9	59.9
S3	**√**	**√**			95.7	69.3	60
S4	**√**		**√**		96.1	70.2	60
S5	**√**			**√**	95.8	69.5	59.9
S6	**√**	**√**	**√**		95.6	70	60
S7	**√**		**√**	**√**	95.4	66.2	60
S8	**√**	**√**		**√**	95.8	69.1	59.9
S9	**√**	**√**	**√**	**√**	96.4	70.8	60

**Table 6:** Experimental results of the ablation study.

Model	mAP50/%	mAP50:95/%	P/%	R/%	Params/M	GFLOPs	FPS
RT-DETR	95.7	70	97.5	93.9	19.87	56.9	119
A	96.3	70.5	97.7	94.1	15.45	51	133
B	96.4	70.8	97.5	94.8	20.5	60	110
C	96.2	70.7	97.5	94.9	20.7	57.8	125
A + B	96.8	70.7	97.4	95.2	16.1	54.1	110
A + C	96.4	69.4	97.8	93.6	16.3	51.9	133
B + C	96.3	71.7	97.8	94.4	21.3	60.9	122
A + B + C	96.9	71.7	97.9	95.5	17	55	119

**Table 7:** Accuracy for Different Object Scales in Module Ablation Studies.

Model	APS/%	APM/%	APL/%
RT-DETR	61.7	74.1	76.9
A	62.4	74.4	77.3
B	62.4	74.6	78.4
C	63.2	74.1	77.7
A + B	62.6	75.4	76.9
A + C	61.6	74.1	74.7
B + C	62.8	76.2	79.5
A + B + C	62.9	76.4	78.6

**Table 8:** Experimental data of mainstream algorithm comparison.

Model	mAP50/%	mAP50:95/%	Params/M	GFLOPs	FPS
RT-DETR	95.7	70	19.87	56.9	119
YOLOv8m	92.8	66	25.86	79.1	153.8
YOLOv11m	92.1	66	20.05	68.2	151.5
YOLOv12m	91.7	65.2	20.1	67	145
SFA-DETR	96.9	71.7	17	55	119

**Table 9:** Comparison of Generalization Experiments.

Dataset	Model	mAP50/%	mAP50:95/%
Det-Fly-simple	RT-DETR	97.2	64.3
	SFA-DETR	97.5	64.7
Det-Fly-complex	RT-DETR	95.7	60.5
	SFA-DETR	97	63.7
Anti2	RT-DETR	75.4	38.5
	SFA-DETR	76.6	41.6

## Data Availability

The original contributions presented in this study are included in the article. Further inquiries can be directed to the corresponding author.
